# Rare Occurrence of Prosthetic Knee Septic Arthritis Due to Streptococcus viridans in the Background of a Dental Procedure

**DOI:** 10.7759/cureus.5980

**Published:** 2019-10-24

**Authors:** Tikal Kansara, Monica Pernia, Yoojin Kim, Mohammad Saeed

**Affiliations:** 1 Internal Medicine, New York Medical College - Metropolitan Hospital Center, New York, USA

**Keywords:** streptococcus viridans, septic arthritis

## Abstract

The American Academy of Oral Medicine, American Dental Association (ADA), in conjunction with the American Academy of Orthopedic Surgeons (AAOS) and the British Society for Antimicrobial Chemotherapy, advises against the universal use of antimicrobial prophylaxis prior to dental procedures for the prevention of prosthetic joint infection (PJI). Here, we discuss the case of a patient with PJI in the background of periodontal scaling, which was done a week prior to presentation to the hospital. The PJI occurred with Streptococcus (S.) viridans, a rare organism for PJI but a common oral commensal. As the number of prosthetic joint surgeries are increasing and more data become available, prophylactic antibiotics might be considered to prevent PJI, especially in high-risk patients.

## Introduction

Septic arthritis remains a medical emergency despite advances in antimicrobial and surgical therapy. Although the rate of prosthetic joint infection (PJI) is low after total knee arthroplasty (TKA), with a reported rate of 0.3% - 1.7%, the number of patients with PJI is increasing due to the increase in TKA surgeries [1-3]. In adults, Staphylococcus aureus is the most common bacterial source of PJI, found in almost 40% - 60% of cases, followed by beta-hemolytic streptococci and Enterococci [4-5]. Viridans streptococci are rarely associated with PJI [4]. We discuss an unusual case of PJI due to the viridans group.

## Case presentation

A 64-year-old female presented with a three-day history of left knee pain and swelling along with high-grade fever (102.6 F) and chills. She had TKA performed eight years prior to her presentation, followed by a total of three revisions; the last revision was three weeks before the current hospital visit. She had periodontal scaling one week ago. Co-morbidities included hypertension (for five years), diet-controlled diabetes mellitus type 2 (for 10 years), chronic obstructive pulmonary disease, chronic hepatitis C, and opioid dependence. Her home medications included aspirin 81 mg, atorvastatin 10 mg, and methadone 35 mg orally daily.

On the initial presentation, the patient’s temperature was 102.6 F, pulse was 122 beats per minute, respiratory rate was 20 per minute, and blood pressure was 125/85 mmHg. Physical examination was normal except for the left knee, which was diffusely erythematous, swollen, warm, and tender.

Investigations

**Figure 1:**
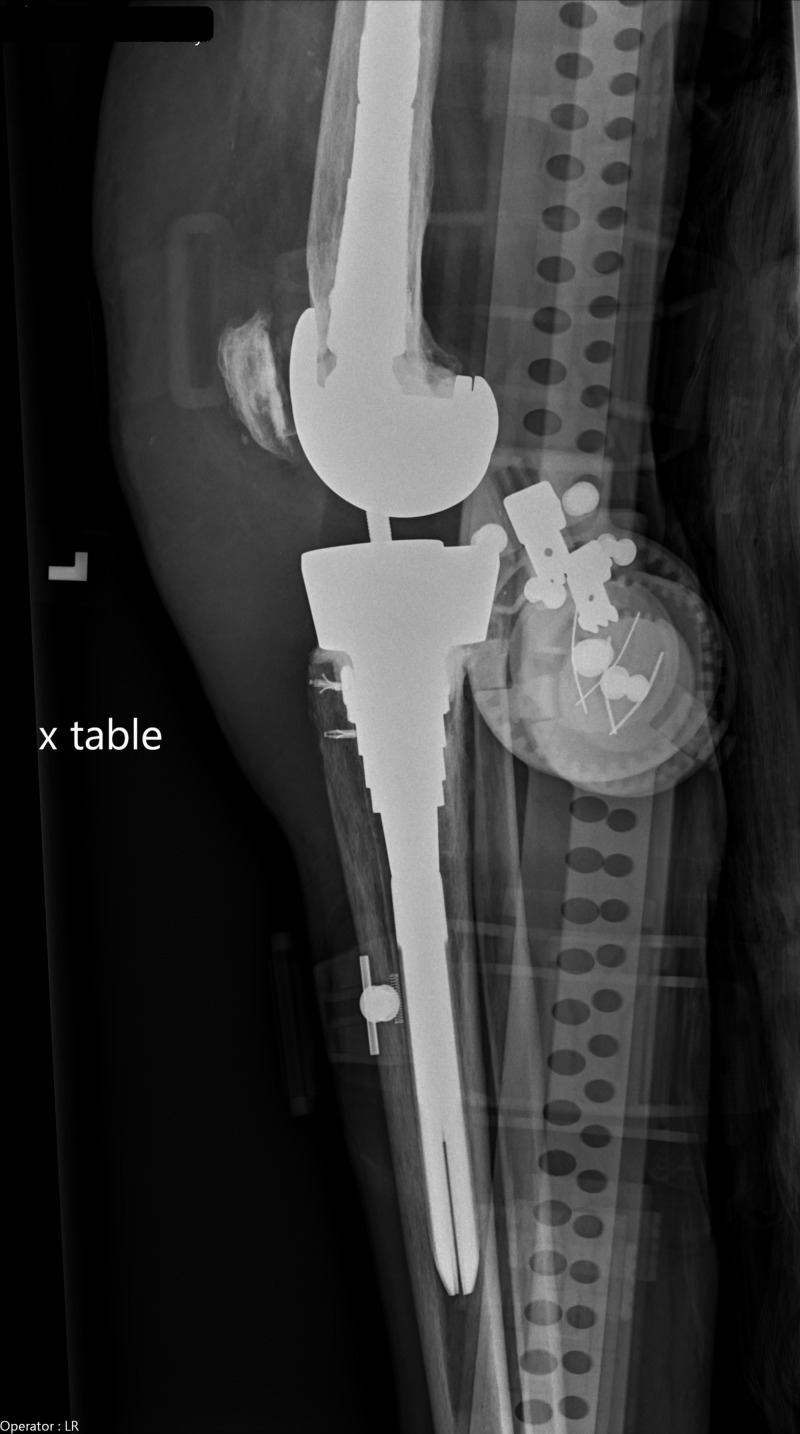
Dx left knee

Her laboratory investigations showed a total leukocyte count of 11.44 cells per cubic millimeter with neutrophilia of 93.58%. C-reactive protein (CRP) was elevated at 15 milligrams per deciliter. Left knee X-ray was suggestive of soft tissue swelling with left knee prosthesis in a satisfactory position without evidence of fracture (Figure 1). Left knee joint arthrocentesis was grossly cloudy in appearance with a white blood cell (WBC) count of 52,800 cells per cubic millimeter, 93% neutrophils, and red blood cells (RBCs) of 177,000 cells per cubic millimeter. Crystal analysis showed calcium pyrophosphate deposition (CPPD). Blood cultures were negative for both aerobic and anaerobic organisms. Synovial fluid culture grew Streptococcus (S.) viridans sensitive to tetracycline, vancomycin, levofloxacin, and intermediate sensitivity to penicillin.

Taking into consideration the patient’s recent dental procedure and synovial fluid analysis showing WBCs and neutrophilia, a diagnosis of prosthetic joint infection was established. Risk factors for septic arthritis also included CPPD and diabetes mellitus. As the organism grew on the second day of culture and was sensitive to routine antibiotics, further testing like 16S rRNA sequencing was deferred.

Treatment

The patient was initially started on vancomycin (1 gm; adjusted with serial vancomycin levels) and piperacillin-tazobactam (3.375 gm) and then switched to fluoroquinolones (levofloxacin 750 mg) as per the synovial fluid culture report. She underwent irrigation and debridement of the left knee and exchange of polyethylene with the retention of the left knee prosthesis. The culture of necrotic and inflamed fibro-connective tissue obtained during irrigation and debridement was negative for any growth. She completed a total of six weeks of antibiotics.

Outcome and follow-up

The patient regularly follows up in the clinic without any further significant events.

## Discussion

The common organisms in prosthetic joint infections are Staphylococcus and beta-hemolytic streptococci [6-9]. Viridans group streptococci are, however, a rare cause of septic arthritis. The S. viridans group is a heterogeneous group of organisms, which consists of 26 species [10-11]. The viridans group is part of normal commensal flora and has low virulence. The infection usually occurs on a previously injured focus [10-12]. A comprehensive review of records does not mention viridans group Streptococcus as one of the etiological agents for septic arthritis [11,13]. However, isolated cases are discussed, involving joints, mainly including the knee, sternoclavicular, acromioclavicular, sacroiliac, and spondylodiscitis [11-18]. Our patient had an infection of the prosthetic knee joint in the background of dental work-up.

Microscopic analysis and culture of synovial fluid are fundamental diagnostic tools for diagnosis. The cytology of synovial fluid usually shows white blood cells of 100,000 cells per cubic millimeter. However, in some cases, cell counts can be less than 25,000 cells per cubic millimeter; as in our case, which does not rule out the possibility of septic arthritis. In knee arthroplasty, a synovial fluid leukocyte count of more than 17000 had a 94% sensitivity and specificity of 88% for prosthetic joint infection [11,19]. The gold standard for diagnosis is the culture of aspirate fluid or prosthetic joint. Other tests to isolate organism is to isolate sterile samples like synovial fluid into BACTEC (Becton, Dickinson and Company, New Jersey, US) system or pediatric isolator tubes. The 16S rRNA gene sequence analysis is useful for sub-classification.

The choice of treatment is antibiotics. There is no one universal antibiotic regimen that has an advantage over the other based on one systematic review and meta-analysis of antibiotic treatment for joint infections [11,20]. The choice of antibiotic is based on culture and sensitivity. In general, S. sanguinis is sensitive to ceftriaxone, S. mitis to clindamycin, and S. anginosus to both ceftriaxone and clindamycin [1]. Our patient’s sensitivity report showed S. viridans sensitive to tetracycline, vancomycin, and levofloxacin. The removal of purulent material can be achieved either surgically or through closed needle aspiration. Our patient underwent irrigation and debridement of the left knee and exchange of polyethylene with the retention of left knee prosthesis. Overall, the duration of antibiotics should depend on the acute condition, co-morbidities of the patient, and a multiple of allied factors, but, in general, at least three weeks of antibiotics is recommended. Our case was unique from all other reported cases as the patient had multiple revisions of the prosthetic joint, which, in itself, acts as a nidus of infection and had a dental procedure a week ago. She did not receive any prophylactic antibiotics prior to the dental procedure. The organism Streptococcus viridans is native to the oral cavity, which was found in the joint fluid aspirate.

## Conclusions

Although rare, streptococcus viridans is a causative organism for prosthetic joint infection. It occurs commonly in cases with recent procedures, especially of an oral cavity. Our patient had multiple revisions of the prosthetic joint and was diabetic. Even though not recommended, prophylactic antibiotics might be considered for patients with prosthetic joints undergoing a dental procedure, especially those with poor dental hygiene and in an immunocompromised condition. Studies with a large number of patients are required to evaluate, in detail, the cause of S. viridans PJI, standardize the line of management, prevent the infection, and define recommendations for prophylactic antibiotics.
